# Enhanced Thermal Safety of Hydrophobic SiO_2_ Aerogels Through Introduction of Layered Double Oxides

**DOI:** 10.3390/gels10120844

**Published:** 2024-12-20

**Authors:** Lei Xu, Guanhua Sun, Jiahui Chen, Xiaoxu Wu, Min Hu, Fang Zhou, Zhi Li

**Affiliations:** 1 Shenyang Fire Science and Technology Research Institute of MEM, 218-20 Wendg Road, Shenyang 110034, China; 2 School of Resource and Safety Engineering, Central South University, Changsha 410083, China

**Keywords:** hydrophobic silica aerogel, layered double oxides, thermal safety, structure

## Abstract

This research enhances the thermal safety of hydrophobic silica aerogel (HSA) by integrating layered double oxides (LDOs). XRD and FTIR confirm that the introduction of LDOs does not affect the formation of SA. The LDO/SA composites demonstrate a low density (0.14–0.16 g/cm^3^), low thermal conductivity (23.28–28.72 mW/(m·K)), high porosity (93.4–96.1%), and a high surface area (899.2–1006.4 m^2^/g). The TG-DSC results reveal that LDO/SA shows enhanced thermal stability, with increases of 49 °C in the decomposition onset temperature and 47.4 °C in the peak decomposition temperature. The gross calorific value of LDO/SA-15% (with 15 wt% LDO) exhibits a 23.9% reduction in comparison to that of pure SA. The decrease in gross calorific value, along with improved thermal stability, indicates a boost in the thermal safety characteristics of the LDO/SA composites. This study demonstrates that incorporating LDOs enhances the thermal safety of HSA, while preserving its superior performance, thus broadening its potential applications in thermal insulation.

## 1. Introduction

Silica aerogel is a porous, inorganic material characterized by the cross-linking of nanoparticles. Its distinctive three-dimensional porous structure imparts several remarkable attributes, including very low thermal conductivity (10–30 mW/(m·K)), ultra-low density (0.003–0.100 g/cm^3^), and extremely high porosity (~99.8%) [1,2,3]. These exceptional properties enable the use of silica aerogel across various applications, including adsorption [4,5], thermal insulation [6,7], catalyst support [8,9], acoustic absorption [10], and aerospace [11]. In practice, the nanoporous network structure is susceptible to water infiltration, which can compromise the gel network and degrade its thermal insulation performance. To address this issue, Si-OH groups on the backbone can be replaced with Si-R groups [12], enhancing hydrophobicity. Nevertheless, the incorporation of organic groups through surface modification may compromise its thermal stability and present potential thermal risks, thereby restricting the use of HSA in insulation applications [13]. To date, various researchers have investigated the approaches aimed at improving the thermal safety of HSA and mitigating its related thermal hazards.

For example, He et al. verified that the heat treatment of hydrophobic methyl-modified SiO_2_ aerogels at various temperatures significantly enhanced their thermal stability [14]. Wu et al. examined how modifiers with different amounts of hydrophobic methyl groups affect the thermal stability of silica aerogels [15]. Li et al. found that incorporating Mg(OH)_2_ and Al(OH)_3_ significantly inhibit the thermal decomposition of SiO_2_ aerogels, with magnesium hydroxide exhibiting a more pronounced effect [16]. However, customized heat treatments not only complicate the process, but also increase energy consumption. Furthermore, the current additives enhance the thermal stability of HSA, while simultaneously increasing its density and thermal conductivity, which ultimately undermines its excellent insulation properties.

In recent times, layered double hydroxides (LDHs) have drawn significant attention for their beneficial properties, including eco-friendliness and exceptional thermal stability. The poor compatibility of LDHs within the polymer matrix adversely affects their flame-retardant properties. However, LDOs produced through heat treatment possess a higher specific surface area and enhanced surface reactivity [17], which can effectively address the issue of poor dispersion. LDOs consist of mixed metal oxides and are commonly utilized in photocatalysis [18] and organic adsorption applications [19]. As is known, the incorporation of metal oxides can enhance thermal safety [20,21], and there may be synergies between different metal oxides; however, the application of LDOs in the study of the thermal safety of HSA has not yet been reported.

Hence, in this study, LDOs were doped into HSA for the first time, and the influence of LDO doping on the microstructure, fundamental physicochemical properties, and thermal stability of LDO/SA were comprehensively investigated. The LDO/SA composites synthesized in this research endeavor manifested enhanced thermal safety characteristics when contrasted with those of pure silica aerogel. This study unequivocally validates the feasibility and practicality of employing LDOs as an effective means to augment the thermal safety of HSA. Consequently, it broadens the spectrum of potential applications of HSA in diverse fields as a promising thermal insulation material.

## 2. Results and Discussion

### 2.1. Microstructure

**Figure 1:**
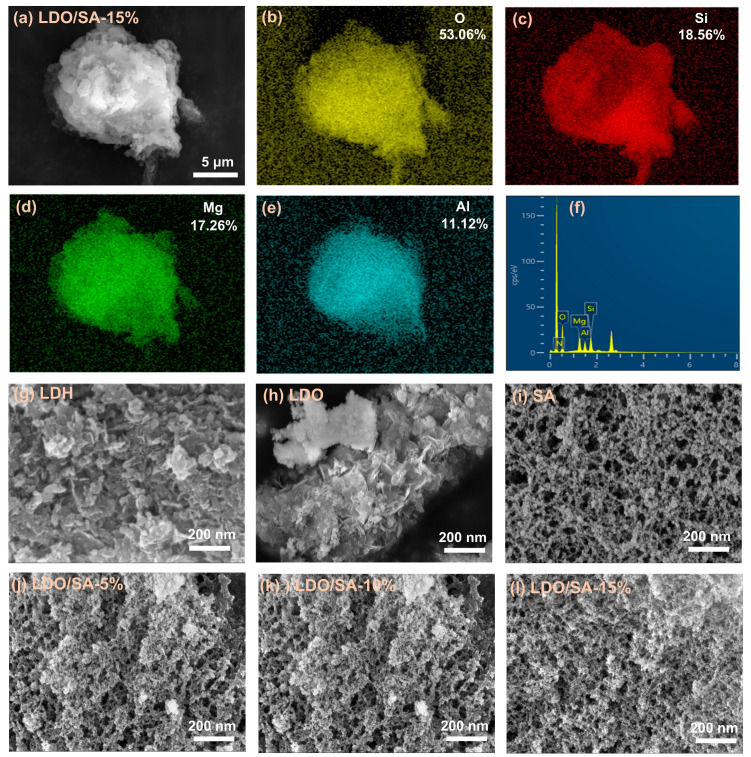
Microstructures of LDO/SA-15% (**a**), EDS spectra of LDO/SA-15% (**b**–**f**), and microstructures of LDH (**g**), LDOs, (**h**) and LDO/SA (**i**–**l**).

Figure 1a presents the microstructure of LDO/SA. As can be seen, the clustered structure of SA particles and the layered structure of LDOs are intertwined in the SEM image of LDO/SA. To further confirm the successful synthesis of LDO/SA, the elemental distribution of LDO/SA was analyzed by EDS, as shown in Figure 1b–f. The magnesium (Mg) and aluminum (Al) elements are derived from the LDOs. The silicon (Si) element comes from SA. Both the LDOs and SA contribute to the oxygen (O) element. From the EDS spectra, the distribution regions of Si, Mg, Al, and O overlap with each other. This indicates that the LDOs are successfully doped to synthesize LDO/SA.

Figure 1g–h provides representations of the microstructures of both LDH and the LDOs. It can be seen that LDH consists of many stacked nanosheets, and a stacking phenomenon likely related to electrostatic forces, van der Waals’ forces, and hydrogen bonding between the metal cationic platelet layers and the interlayer ions [22]. The morphology of LDH is more compact than that of the LDOs, which is produced through calcination at 500 °C. During calcination, interlayer anions and water molecules are removed from LDH, disrupting its layered structure and resulting in the formation of LDOs.

Figure 1i–l presents the microstructures of pure SA, as well as the LDO/SA composites that contain varying amounts of LDO. Obviously, pure silica aerogel exhibits a unique silica skeleton network structure, wherein the secondary particles are interconnected to create a three-dimensional mesoporous architecture resembling a ‘pearl chain’. Notably, with the further increase in the doping amount of LDO, the pore size of LDO/SA decreases, and the agglomeration of particles occurs, which suggests that the SiO_2_ skeleton undergoes a slight collapse.

**Figure 2:**
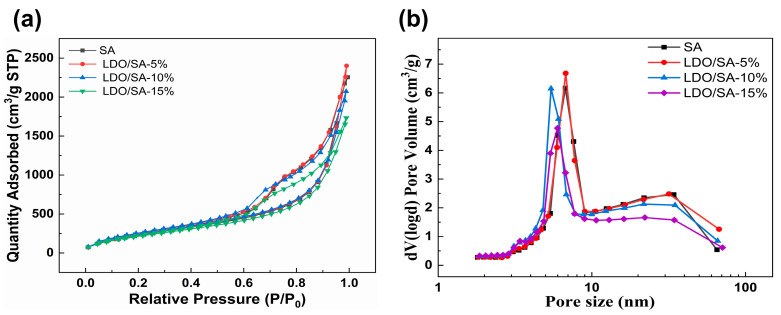
N_2_ adsorption isotherms (**a**) and pore size distribution (**b**) for SA and LDO/SA.

Nitrogen adsorption isotherms allow for the analysis of the internal pore structure and pore shape of materials. As shown in Figure 2, all the LDO/SA composites exhibit nitrogen adsorption isotherms resembling that of pure SA. Based on the IUPAC classification [23], all the nitrogen adsorption isotherms are classified as typical type IV isotherms, which indicates that the LDO/SA composites have a microscopic mesoporous structure as well as pure silica aerogel, while the presence of the H3-type hysteresis loop confirms the existence of slit-like pores among the internal particles [24]. The disappearance of the saturation adsorption platform in the vicinity of a relative pressure of 1.0 implies the presence of macropores and voids within the LDO/SA, as can be discerned from the pore size distribution curves [25]. Consequently, the incorporation of LDOs did not have a substantial impact on the mesoporous characteristics of the LDO/SA composites. Moreover, LDO/SA possesses the largest volume of nitrogen when the doping level of LDOs is 5 wt%. As the LDO doping level further increases, the nitrogen adsorption of LDO/SA gradually decreases, which is related to the collapse of the silica skeleton caused by excessive LDO doping, so ultimately a smaller pore volume is formed.

Figure 2b illustrates the pore size distributions for both pure silica aerogel and the LDO/SA composites. The pore size distribution of LDO/SA closely resembles that of pure silica aerogel, which is mainly concentrated between 5 and 10 nm. When the LDO doping total is 5 wt%, the peaks of LDO/SA pore size distribution are narrower and higher, suggesting a more homogeneous and concentrated internal pore size distribution, and as LDO doping continuously increases, the most likely aperture of LDO/SA shifts becomes smaller. LDO/SA and SA exhibit a mesoporous structure, indicating that LDO doping has a minimal impact on the microscopic pore characteristics of silica aerogels.

Table 1 presents the comprehensive pore parameters, which encompass the pore volume, the average pore size, and the specific surface area. Incorporating LDOs into LDO/SA increases its specific surface area, primarily because LDOs derived from the calcination of LDH have a higher specific surface area [17]. Furthermore, LDOs demonstrate a nitrogen adsorption capacity, while occupying some of the pore space within the silica framework. A moderate amount of LDO doping facilitates the formation of smaller and more uniform pores, which also promotes a larger specific surface area. Significantly, both the total pore volume and the average pore size of LDO/SA initially increase before subsequently decreasing. These findings may be explained by the presence of a limited number of LDO nanosheets that either occupy or obstruct some pores, resulting in an increase in the average pore size. These findings may be due to the limited number of LDO nanosheets that either fill or block certain pores, leading to an increase in the average pore size. However, the over-doping of LDO disrupts the skeleton structure of silica aerogel and forms smaller or confined pores, which is aligned with the SEM findings.

However, the BJH method has some limitations, so we calculated *V_pore_* and *D_pore_* using Equations (1) and (2), respectively. The calculated *V_pore_* and *D_pore_* values are larger, suggesting the presence of pores in the LDO/SA skeleton that exceed the nitrogen adsorption test’s characterization limits [26]. Additionally, the actual nitrogen adsorption–desorption test process also influences the structure of the silica aerogel skeleton to a certain extent.

**Table 1:** Pore parameters of SA and LDO/SA.

Sample	BET Surface Area (m^2^/g)	Total Pore Volume ^a^ (cm^3^/g)	Average Pore Size ^b^ (nm)	*V_pore_* ^c^ (cm^3^/g)	*D_pore_*^d^ (nm)
SA	967.4	3.5	14.4	10.1	41.8
LDO/SA-5%	981.4	3.7	15.1	11.3	46.1
LDO/SA-10%	1006.4	3.2	12.7	9.0	35.8
LDO/SA-15%	899.2	2.7	11.9	6.6	29.4

^a, b^ Pore volume and average pore diameter were calculated from nitrogen desorption. Uncertainties: BET surface area around 50 m^2^/g; pore volume and average pore diameter, 5% relative [27]. ^c, d^ *V_pore_* and *D_pore_* were obtained using Equations (1) and (2) with 10% relative uncertainty [28].

### 2.2. Density, Porosity, and Thermal Conductivity

**Figure 3:**
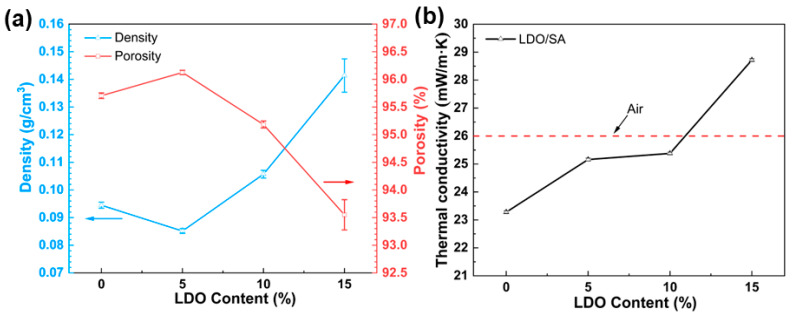
Density and porosity (**a**), along with thermal conductivity (**b**) of SA and LDO/SA.

Figure 3 presents the porosity, tap density, and thermal conductivity of the LDO/SA composites at various LDO contents. A slight decrease in the density of LDO/SA powder is observed with the incorporation of 5% wt LDO, likely caused by electrostatic repulsion that expands the particle gaps in the composites. With the LDO content increasing, more LDO particles fill the silica aerogel pores, and the higher density of the LDOs themselves counteracts electrostatic repulsion, leading to a continuous increase in LDO/SA density. In addition, the change in porosity shows an opposite trend to that of density. The decrease in porosity is related to the pore space occupied by the LDOs in the aerogel skeleton and the collapse of the porous skeleton due to over-doping. However, LDO/SA continues to exhibit the fundamental attributes of low density and high porosity.

As illustrated in Figure 3b, the LDO/SA composites’ thermal conductivity increases from 23.3 mW/(m·K) to 28.7 mW/(m·K) as the LDO content rises from 0% to 15%. This is due to the interaction between LDO doping and the aerogel skeleton, which enhances the heat transfer pathway. In addition, excessive doping of LDOs causes the collapse of the aerogel skeleton, significantly increasing thermal conductivity. Notably, when the content of LDO is 10%, the thermal conductivity of LDO/SA remains lower that of static air (about 26 mW/(m·K)), demonstrating an excellent thermal insulation performance.

### 2.3. Surface Chemistry and Hydrophobicity

**Figure 4:**
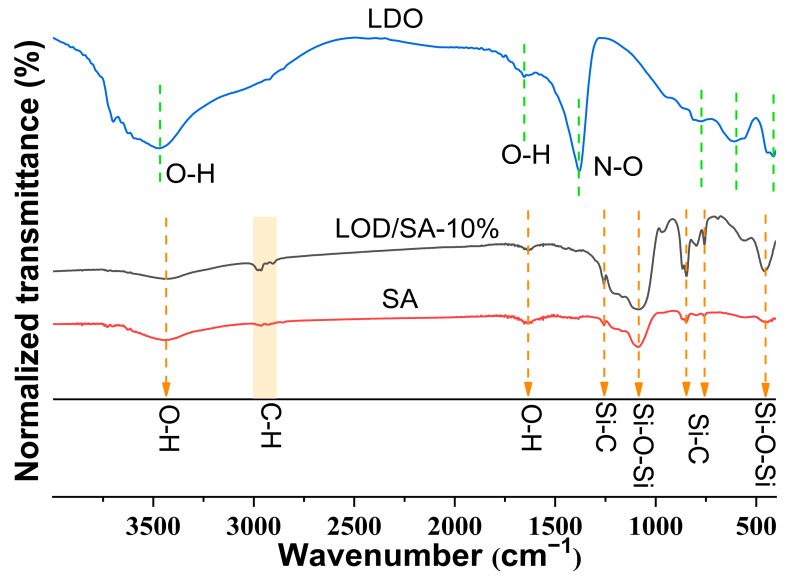
FTIR spectra of LDO, LDO/SA-10%, and pure SA.

Figure 4 illustrates the FTIR spectra of pure silica aerogel (SA), the LDOs, and LDO/SA. In the case of SA, the FTIR spectrum shows asymmetric stretching and deformation vibrations of the Si-O-Si bonds at 1084 cm^−1^ and 453 cm^−1^, respectively [29]. The peaks around 3437 cm^−1^ and 1635 cm^−1^ are attributed to the stretching and bending vibrations of water molecules that are physically adsorbed onto the surface [30].

The absorption peaks between 2980 and 2900 cm^−1^ correspond to the symmetric and asymmetric stretching vibrations of C-H bonds, whereas those at 1260 cm^−1^, 845 cm^−1^, and 756 cm^−1^ are associated with Si-C bonds [14]. These results confirm that -CH_3_ groups were effectively introduced into the silicon skeleton through surface modification [31].

In the FTIR spectra of the LDOs, the 3468 cm^−1^ peak is associated with OH stretching vibrations from residual hydroxyl groups, while the band at 1655 cm^−1^ corresponds to the bending vibrations of interlayer water molecules [32]. The 1380 cm^−1^ peak related to N-O stretching in nitrate [33] indicates that the interlayer anions were not completely removed after calcination. The absorption bands within the 400–900 cm^−1^ region are linked to the stretching vibrations of M-O-M and O-M-O, where M denotes Mg^2^⁺ and Al^3^⁺ [33,34].

Evidently, no new chemical bonds were observed in the IR spectra of LDO/SA. The LDO/SA apparent absorption peak is a simple overlay of the LDO and SA absorption peaks, which indicates that the two components are physically bonded.

**Figure 5:**
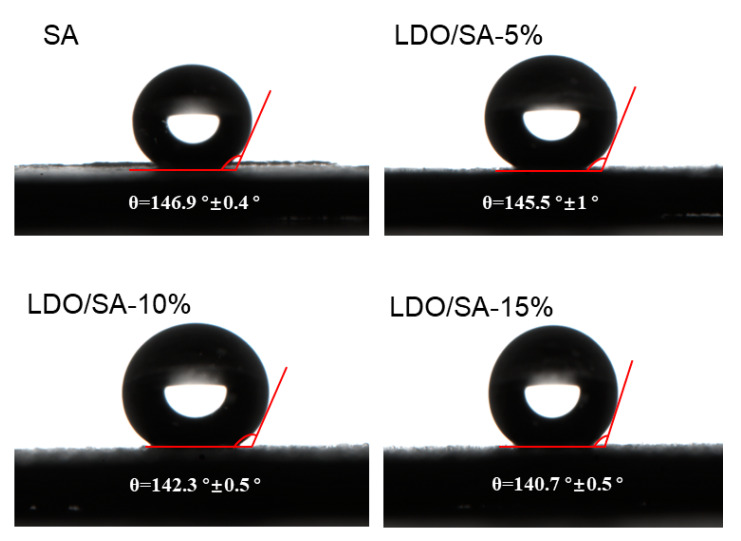
Contact angles of SA and LDO/SA.

The hydrophobicity of both SA and LDO/SA was assessed by measuring their contact angles, as illustrated in Figure 5. Pure SA demonstrated pronounced hydrophobicity, with a contact angle close to 146.9°. However, increasing LDO doping led to a slight reduction in the hydrophobicity of the LDO/SA composites. The contact angle of LDO/SA decreased to 140.7° when the LDO amount was 15 wt%, indicating that the hydrophobicity of the composites was reducing. The deterioration of hydrophobic properties is likely attributed to the introduction of residual hydrophilic hydroxyl groups onto the SA surface by LDO, as evidenced by the FTIR spectra. Nonetheless, the addition of LDOs does not significantly alter the overall hydrophobicity, as all the samples retain contact angles exceeding 140°, indicating strong hydrophobic characteristics.

**Figure 6:**
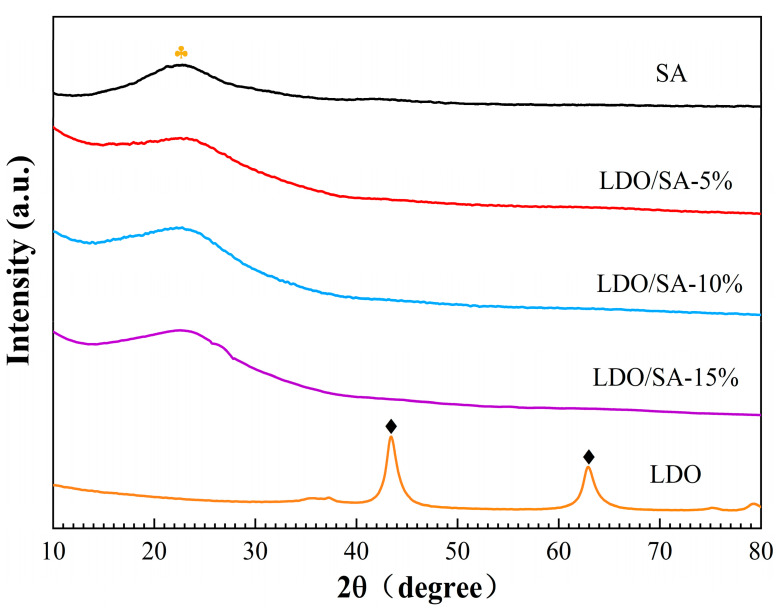
XRD patterns of SA, LDOs, and LDO/SA.

The XRD patterns of the LDOs, SA, and LDO/SA are presented in Figure 6. Pure SA exhibits a broad scattering peak at 2θ = 22.46°, suggesting an amorphous structure [14]. Additionally, the XRD patterns of LDO reveal characteristic diffraction peaks attributed to MgO (200) and (220) crystalline phases at 2θ = 43.4° and 63°, respectively. This suggests that LDH decomposition occurred during calcination, leading to the formation of bimetallic oxides. Notably, the XRD patterns of the LDOs did not exhibit diffraction peaks corresponding to the crystalline phase of Al_2_O_3_, possibly due to the substantial dispersion of Al^3^⁺ on their surface [35,36]. The XRD pattern of LDO/SA shows clear similarities to that of pure SA, with no new characteristic diffraction peaks observed. This indicates that the doping of LDO does not significantly affect the physical phase structure of SA.

### 2.4. Thermal Safety

**Figure 7:**
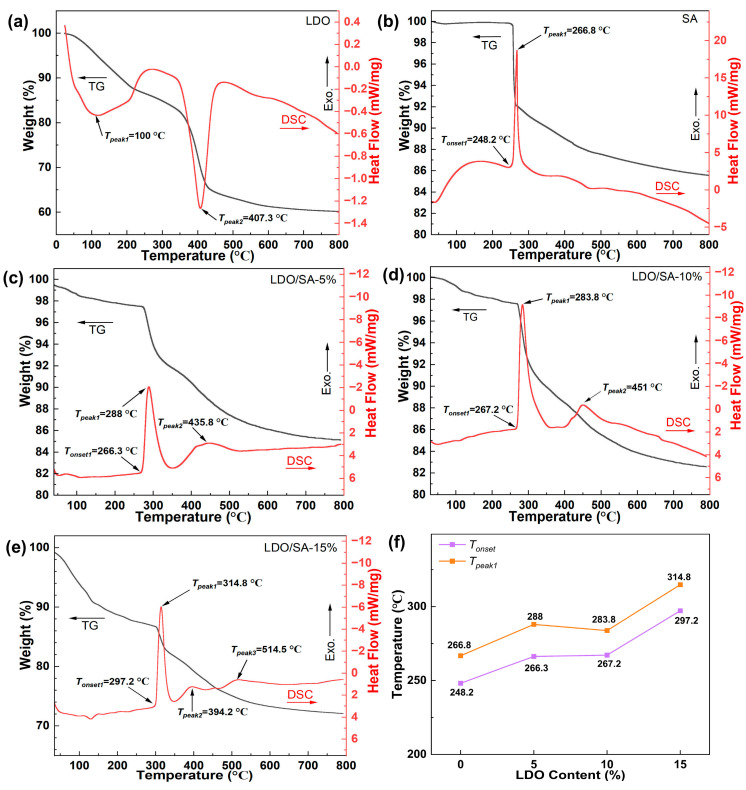
TG-DSC curves of LDOs (**a**), SA, (**b**), and LDO/SA (**c**–**e**); detailed LDO/SA composite DSC data (**f**).

The thermal stability of the LDOs, SA, and LDO/SA was evaluated and is presented in Figure 7. The TG-DSC curves of LDH are depicted in Figure 7a, demonstrating that the LDOs underwent thermal decomposition in two distinct phases.

The first stage, occurring around 100 °C, exhibits a heat absorption peak associated with the removal of physically adsorbed water molecules. The second phase, around 400 °C, corresponds to a mass loss of 21.7% due to the decomposition of residual interlayer nitrate anions and hydroxyl groups in the LDOs.

Figure 7b illustrates the TG-DSC curves of pure SA, indicating that its thermal degradation process also occurred in two stages. The initial stage involves minor mass loss, attributed to the heat-absorbing volatilization of a small amount of residual solvent and water present in pure SA [37]. In the second stage, significant weight loss occurs in the pure SA sample as the temperature exceeds 252.2 °C. An exothermic peak at 266.8 °C on the DSC curve corresponds to a 14.2% mass reduction, likely due to the pyrolysis of Si-CH_3_ groups on the silicon framework, which also destroys the overall hydrophobicity [14].

The TG-DSC analysis of LDO/SA is shown in Figure 7c–e, revealing a two-stage weight loss process for the composite. For the TG-DSC curve, we can still divide the weightlessness process of the LDO/SA composite into two stages. In the initial phase, mass reduction is primarily due to the evaporation of residual solvents and water contained within the pores of the LDO/SA, as well as the removal of the hydroxyl groups remaining in the LDOs. Additionally, this stage demonstrates an increased mass loss with higher LDO doping levels. The second phase, which involves the largest mass loss, is driven by the exothermic decomposition of Si-CH_3_ groups introduced via surface modification, along with the condensation of OH groups [31], which also contributes to the reduction in mass.

*T_onset_* and *T_peak_* reflect the thermal stability of the material, providing insights into its thermodynamic behavior and structural changes. As shown in Figure 7f, the *T_onset_* and *T_peak_*_1_ for pure SA and LDO/SA represent the onset and peak temperatures of Si-CH_3_ thermo-oxidative decomposition.

With an increasing LDO doping content, the *T_onset_* of LDO/SA rises from 248.2 °C to 297.2 °C, and *T_peak_*_1_ lifts from 267.4 °C to 314.8 °C, exceeding those of pure SA by 49 °C and 47.4 °C, respectively. Evidently, this demonstrates a substantial improvement in the thermal stability of HSA due to LDO doping. This enhancement is likely due to the decomposition of residual hydroxyl groups within the LDOs, which absorb heat and effectively lower the internal temperature. Furthermore, the LDOs act as an effective thermal barrier, minimizing internal heat transfer.

**Figure 8:**
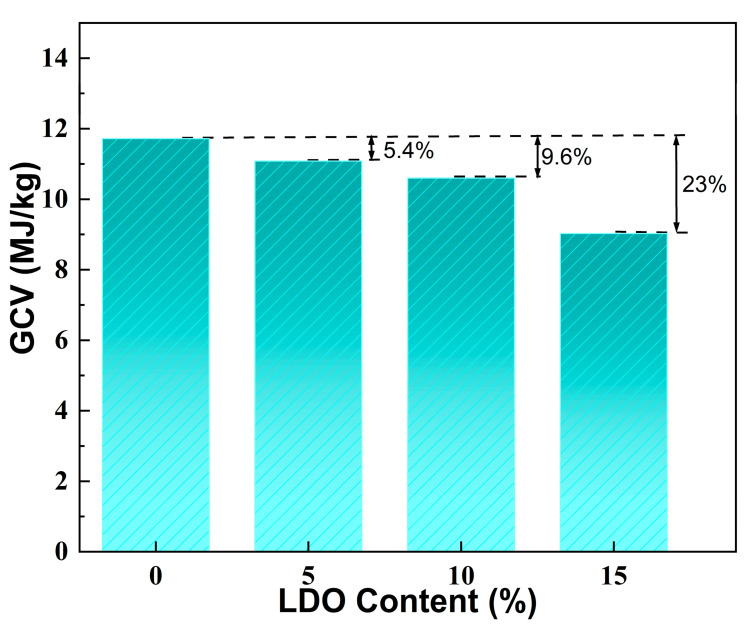
GCV of SA and LDO/SA composites.

The gross calorific value (GCV) represents the amount of heat released during the complete combustion of a material and is a crucial measure of its thermal safety. Figure 8 illustrates the GCV values for SA and the LDO/SA composites. As illustrated in the figure, SA exhibits a GCV of 11.7 MJ/kg, while the GCV of LDO/SA exhibits a decreasing trend as the LDO doping content increases. At 15 wt% LDO doping, the GCV of LDO/SA declines to 9.0 MJ/kg, reflecting a 23% reduction compared to that of SA. This reduction is primarily due to the heat-absorbing decomposition of residual hydroxyl groups in the LDOs, which lowers the system’s temperature. Additionally, the LDOs’ capacity to function as a physical barrier further impedes combustion. These findings presented above demonstrate that LDO doping substantially enhances the thermal safety of HSA.

## 3. Conclusions

Herein, we achieved the successful synthesis of LDO/SA composites through the doping of LDOs into HSA, with an emphasis on augmenting their thermal safety. It was observed that when contrasted with that of pure SA, the microstructure of LDO/SA exhibited minimal alterations, possessing characteristics such as low density, low thermal conductivity, and outstanding hydrophobicity. The investigation disclosed that LDO doping effectively mitigated the pyrolysis of Si-CH3 on the silica backbone, consequently enhancing the thermal stability of LDO/SA. Additionally, LDO doping led to a significant reduction in the gross calorific value (GCV) of HSA. The improvement in thermal safety can be ascribed to the endothermic and thermal barrier effects of LDOs. This study presents a facile methodology for enhancing the thermal safety of HSA, which is beneficial for broadening the application scope of silica aerogel materials in thermal insulation across a diverse range of fields.

## 4. Materials and Methods

### 4.1. Raw Materials

Ethyl silicate (TEOS), ethanol (EtOH, 99.7%), and n-hexane (97.0%) were sourced from Sinopharm Chemical Reagent Co., Ltd. (Shanghai, China). For the catalytic processes, nitric acid (HNO₃, 36–38%) acted as the acid catalyst, while ammonia solution (NH₃·H₂O, 25–28%) functioned as the base catalyst. Other chemicals, including sodium hydroxide (NaOH, 96.0%), trimethylchlorosilane (TMCS, 98%), aluminum nitrate nonahydrate (Al(NO₃)₃·9H₂O, 99.0%), and magnesium nitrate hexahydrate (Mg(NO₃)₂·6H₂O, 99.0%), were obtained from Aladdin Reagent Co., Ltd. (Shanghai, China). High-purity deionized water was generated using an ECO-S ultra-pure water system (ECO-S, HHitech, Shanghai, China) to maintain consistent experimental conditions.

### 4.2. Sample Preparation

**Synthesis of MgAl-LDO:** Magnesium–aluminum-layered double hydroxides (Mg/Al-LDHs) were prepared using a co-precipitation method, followed by calcination to obtain Mg/Al layered double oxides (Mg/Al-LDOs). The specific preparation details are as follows: First, an amount of A mixture of Al(NO_3_)_3_·9H_2_O and Mg(NO_3_)_2_·6H_2_O was dissolved in deionized water and transferred to a three-necked flask under inert gas. A total of 0.8 mol/L NaOH solution was then added dropwise until the pH reached 12, with continuous stirring to ensure homogeneous mixing. The resulting suspension underwent crystallization at 75 °C for 24 h, followed by filtration, and was washing until neutral. The dried precipitate was calcined at 500 °C for 2 h to yield LDO powder.

**Figure 9:**
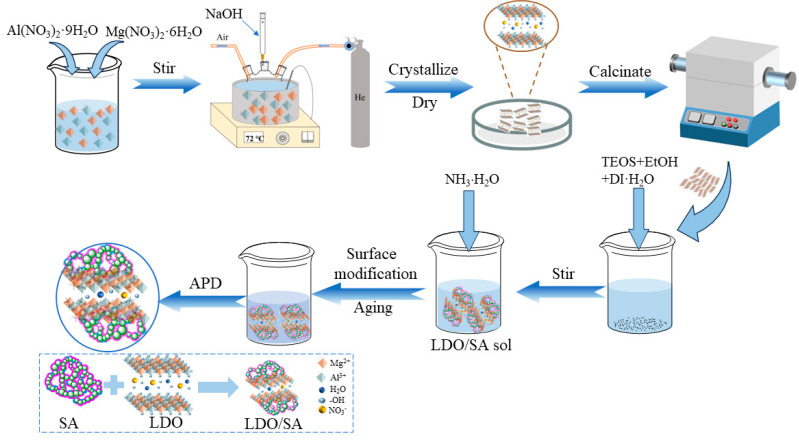
Flowchart of LDO/SA preparation.

**Preparation of LDO/SA Composites:** The entire preparation process is illustrated in Figure 9, which depicts a flowchart of LDO/SA preparation. Initially, specific quantities of ethanol, HNO_3_, TEOS, and deionized water in a volume ratio of 15:0.3:5.75:1 were mixed and hydrolyzed at 45 °C for 12 h. Subsequently, different proportions of LDO (accounting for 0 wt%, 5 wt%, 10 wt%, and 15 wt% of the entire composite) were dispersed into silica solution. This was accomplished by means of mechanical stirring at 4000 revolutions per minute (rpm) for 10 min, followed by ultrasonic dispersion at a frequency of 40 kHz for 30 min. The resulting samples were named SA, LDO/SA-5%, LDO/SA-10%, and LDO/SA-15%, respectively. A total of 0.5 M ammonia solution (NH_3_·H_2_O, aqueous) was then slowly added to hydrolysate and stirred for 3 min. Thereafter, the mixture was maintained at 45 °C to form a gel. Subsequently, the gel was chopped and subjected to solvent exchange using ethanol and hexane successively, with an interval of 12 h between each exchange. Upon the completion of solvent exchange, TMCS was added to the wet gel for surface modification over a period of 12 h. After the modification was finished, the residual TMCS on the surface of the wet gel was washed off using hexane. Finally, the modified wet gel was dried in an oven at 120 °C for 4 h to obtain the LDO/SA composites.

### 4.3. Methods of Characterization

A field-emission scanning electron microscope (SEM, Sigma 300, Zeiss, Oberkochen, Germany) was used to analyze the microstructure of LDO/SA composites, and the crystalline phases were analyzed via X-ray diffraction (XRD, D8 Advance, Bruker, Billerica, MA, USA). Additionally, nitrogen adsorption–desorption measurements were performed using an automatic surface area and porosity analyzer (Quantachrome, AUTOSORB IQ, Boynton Beach, FL, USA) to determine surface area, pore size distribution, and total pore volume. The BET method [38] and the BJH method [39] were applied to calculate specific surface area and pore characteristics, respectively. To further investigate the internal pore structure, we also calculated average pore diameter (*D_pore_*) and pore volume (*V_pore_*) using Equations (1) and (2).

(1) $$V_{pore}=\frac{1}{\rho_{t}}-\frac{1}{\rho_{s}}$$

(2) $$D_{pore}=\frac{4V_{\mathrm{por}e}}{S_{BET}}$$

where *ρ_t_*, *ρ_s_,* and *S_BET_* represent tap density, skeletal density, and the specific surface area obtained by BET method, respectively.

The tap density (*ρ_t_*) of pure SA and the LDO/SA composites was assessed using a tap density meter (ZS-202, Liaoning Instrument Research Institute, Dandong, China). A 10 mL graduated cylinder was subjected to continuous vibration at 300 rpm for 10 min to ensure uniform packing. The porosity of composites was evaluated using Equations (3) and (4).

(3) $$Porosity=\left(1-\frac{\rho_{t}}{\rho_{s}}\right)\times 100\%$$

(4) $$\frac{1}{\rho_{s}}=\frac{c}{\rho_{LDO}}-\frac{1-c}{\rho_{SA}}$$

here, *ρ_s_* represents the skeletal density of the composites, with the LDOs having an approximate density of 2.15 g/cm^3^, and SA of 2.2 g/cm^3^. *c* refers to the mass fraction of LDO in the composite material.

Thermal conductivity measurements were conducted at room temperature using a thermostatic thermal analyzer (TC3000E, XIATECH, Xi’an, China) and the transient hot wire method.

Fourier-transform infrared spectroscopy (FTIR, Nicolet iS50, Thermo Fisher Scientific, Waltham, MA, USA) was performed to determine the chemical groups and bonds present in the composite materials.

The attenuated total reflectance (ATR) method was used for sample preparation, ensuring minimal preparation and accurate data collection. Hydrophobicity was assessed via the drip method, with contact angle measurements using an automatic meter (JC2000D1, Shanghai Zhongchen Instrument, Shanghai, China).

Thermal stability was carried out by thermogravimetric-differential scanning calorimetry (TG-DSC) using a simultaneous thermal analyzer (TG-DSC, STA 449 F3, NETZSCH, Selb, Germany) in an air atmosphere, with a controlled heating rate of 10 °C/min from room temperature to 800 °C. The gross calorific values (GCVs) of silica aerogel (SA) and the LDO/SA composites were measured using an oxygen bomb calorimeter (Yuan Fa AM-C1009, Changsha, China) to quantify the energy released during combustion.

## Data Availability

The original contributions presented in this study are included in the article. Further inquiries can be directed to the corresponding authors.

## References

[1] Wang Y., Li Z., Huber L., Wu X., Huang S., Zhang Y., Huang R., Liu Q. (2020). Reducing the thermal hazard of hydrophobic silica aerogels by using dimethyldichlorosilane as modifier. J. Sol Gel Sci. Technol..

[2] Akhter F., Soomro S.A., Inglezakis V.J. (2021). Silica aerogels; a review of synthesis, applications and fabrication of hybrid composites. J. Porous Mater..

[3] Maleki H., Durães L., Portugal A. (2014). An overview on silica aerogels synthesis and different mechanical reinforcing strategies. J. Non Cryst. Solids.

[4] Tang R., Hong W., Srinivasakannan C., Liu X., Wang X., Duan X. (2022). A novel mesoporous Fe-silica aerogel composite with phenomenal adsorption capacity for malachite green. Sep. Purif. Technol..

[5] Mohammadi A., Moghaddas J. (2015). Synthesis, adsorption and regeneration of nanoporous silica aerogel and silica aerogel-activated carbon composites. Chem. Eng. Res. Des..

[6] Shang L., Lyu Y., Han W. (2019). Microstructure and Thermal Insulation Property of Silica Composite Aerogel. Materials.

[7] Liu Z., Lyu J., Fang D., Zhang X. (2019). Nanofibrous Kevlar Aerogel Threads for Thermal Insulation in Harsh Environments. ACS Nano.

[8] Tai Y., Tajiri K. (2008). Preparation, thermal stability, and CO oxidation activity of highly loaded Au/titania-coated silica aerogel catalysts. Appl. Catal. A Gen..

[9] Amiri T.Y., Moghaddas J., Khajeh S.R. (2016). Silica aerogel-supported copper catalyst prepared via ambient pressure drying process. J. Sol Gel Sci. Technol..

[10] Mazrouei-Sebdani Z., Begum H., Schoenwald S., Horoshenkov K.V., Malfait W.J. (2021). A review on silica aerogel-based materials for acoustic applications. J. Non Cryst. Solids.

[11] Bheekhun N., Talib A.R.A., Hassan M.R. (2013). Aerogels in Aerospace: An Overview. Adv. Mater. Sci. Eng..

[12] Li Z., Zhao S., Koebel M.M., Malfait W.J. (2020). Silica aerogels with tailored chemical functionality. Mater. Des..

[13] Li Z., Hu M., Shen K., Liu Q., Li M., Chen Z., Cheng X., Wu X. (2024). Tuning thermal stability and fire hazards of hydrophobic silica aerogels via doping reduced graphene oxide. J. Non Cryst. Solids.

[14] He S., Huang Y., Chen G., Feng M., Dai H., Yuan B., Chen X. (2019). Effect of heat treatment on hydrophobic silica aerogel. J. Hazard. Mater..

[15] Wu X., Zhang W., Li Z., Zhang Y., Huang S., Liu Q. (2019). Effects of various methylchlorosilanes on physicochemical properties of ambient pressure dried silica aerogels. J. Nanopart. Res..

[16] Li Z., Huang S., Shi L., Li Z., Liu Q., Li M. (2019). Reducing the flammability of hydrophobic silica aerogels by doping with hydroxides. J. Hazard. Mater..

[17] Lee S.-B., Ko E.-H., Park J.Y., Oh J.-M. (2021). Mixed Metal Oxide by Calcination of Layered Double Hydroxide: Parameters Affecting Specific Surface Area. Nanomaterials.

[18] Abderrazek K., Najoua F.S., Srasra E. (2016). Synthesis and characterization of [Zn–Al] LDH: Study of the effect of calcination on the photocatalytic activity. Appl. Clay Sci..

[19] Yao W., Yu S., Wang J., Zou Y., Lu S., Ai Y., Alharbi N.S., Alsaedi A., Hayat T., Wang X. (2017). Enhanced removal of methyl orange on calcined glycerol-modified nanocrystallined Mg/Al layered double hydroxides. Chem. Eng. J..

[20] Friederich B., Laachachi A., Ferriol M., Ruch D., Cochez M., Toniazzo V. (2010). Tentative links between thermal diffusivity and fire-retardant properties in poly(methyl methacrylate)–metal oxide nanocomposites. Polym. Degrad. Stab..

[21] Kim Y.N., Shao G.N., Jeon S.J., Imran S.M., Sarawade P.B., Kim H.T. (2013). Sol–gel synthesis of sodium silicate and titanium oxychloride based TiO_2_–SiO_2_ aerogels and their photocatalytic property under UV irradiation. Chem. Eng. J..

[22] Sun M., Wang Y., Wang X., Liu Q., Li M., Shulga Y.M., Li Z. (2022). In-Situ Synthesis of Layered Double Hydroxide/Silica Aerogel Composite and Its Thermal Safety Characteristics. Gels.

[23] Rojas F., Kornhauser I., Felipe C., Esparza J.M., Cordero S., Domínguez A., Riccardo J.L. (2002). Capillary condensation in heterogeneous mesoporous networks consisting of variable connectivity and pore-size correlation. Phys. Chem. Chem. Phys..

[24] Sing K. (1985). Reporting physisorption data for gas/solid systems with special reference to the determination of surface area and porosity (Recommendations 1984). Pure Appl. Chem..

[25] Sun Z., Zhao Z., Kong Y., Ren J., Jiang X., Shen X. (2022). Auto-Continuous Synthesis of Robust and Hydrophobic Silica Aerogel Microspheres from Low-Cost Aqueous Sodium Silicate for Fast Dynamic Organics Removal. Gels.

[26] Li Z., Hu M., Shen K., Liu Q., Li M., Wu X. (2024). Low phosphorus-containing hydrophobic silica aerogels with high thermal stability and outstanding flame retardancy. Polym. Degrad. Stab..

[27] Kantor Z., Wu T., Zeng Z., Gaan S., Lehner S., Jovic M., Bonnin A., Pan Z., Mazrouei-Sebdani Z., Opris D.M. (2022). Heterogeneous silica-polyimide aerogel-in-aerogel nanocomposites. Chem. Eng. J..

[28] Ji S., Chen Y., Wang X., Zhang Z., Wang D., Li Y. (2020). Chemical Synthesis of Single Atomic Site Catalysts. Chem. Rev..

[29] Shao Z., Luo F., Cheng X., Zhang Y. (2013). Superhydrophobic sodium silicate based silica aerogel prepared by ambient pressure drying. Mater. Chem. Phys..

[30] Al-Oweini R., El-Rassy H. (2009). Synthesis and characterization by FTIR spectroscopy of silica aerogels prepared using several Si(OR)_4_ and R”Si(OR’)_3_ precursors. J. Mol. Struct..

[31] Wu X., Li Z., Joao G., Zhang Y., Huang S., Liu Q. (2020). Reducing the flammability of hydrophobic silica aerogels by tailored heat treatment. J. Nanopart. Res..

[32] Qin Q., Wang J., Zhou T., Zheng Q., Huang L., Zhang Y., Lu P., Umar A., Louis B., Wang Q. (2017). Impact of organic interlayer anions on the CO_2_ adsorption performance of Mg-Al layered double hydroxides derived mixed oxides. J. Energy Chem..

[33] Ahmed A.A.A., Talib Z.A., Hussein M.Z.B., Zakaria A. (2012). Improvement of the crystallinity and photocatalytic property of zinc oxide as calcination product of Zn-Al layered double hydroxide. J. Alloy Compd..

[34] Wen T., Wu X., Tan X., Wang X., Xu A. (2013). One-Pot Synthesis of Water-Swellable Mg-Al Layered Double Hydroxides and Graphene Oxide Nanocomposites for Efficient Removal of As(V) from Aqueous Solutions. ACS Appl. Mater. Interfaces.

[35] Zhang Z., Xu S., Zeng H., Liao M., Du J., Duan H. (2016). Influence of Calcination Temperature on the Microstructure and Catalytic Performance of Mg/Al Hydrotalcites Catalysts for Alcoholysis of Propylene Oxide. J. Nanosci. Nanotechnol..

[36] Pinthong P., Praserthdam P., Jongsomjit B. (2019). Effect of Calcination Temperature on Mg-Al Layered Double Hydroxides (LDH) as Promising Catalysts in Oxidative Dehydrogenation of Ethanol to Acetaldehyde. J. Oleo Sci..

[37] Bippus L., Jaber M., Lebeau B., Schleich D., Scudeller Y. (2014). Thermal conductivity of heat treated mesoporous silica particles. Microporous Mesoporous Mater..

[38] Brunauer S., Emmett P.H., Teller E. (1938). Adsorption of Gases in Multimolecular Layers. J. Am. Chem. Soc..

[39] Barrett E.P., Joyner L.G., Halenda P.P. (1951). The Determination of Pore Volume and Area Distributions in Porous Substances. I. Computations from Nitrogen Isotherms. J. Am. Chem. Soc..

